# *GSTM1* and *GSTP1* Polymorphisms Affect Outcome in Colorectal Adenocarcinoma

**DOI:** 10.3390/medicina60040553

**Published:** 2024-03-28

**Authors:** Milica Stojkovic Lalosevic, Vesna Coric, Tatjana Pekmezovic, Tatjana Simic, Aleksandra Pavlovic Markovic, Marija Pljesa Ercegovac

**Affiliations:** 1 Clinic of Gastroenterology and Hepatology, University Clinical Center of Serbia, 11000 Belgrade, Serbia; drmilicastojkovic@gmail.com; 2 Faculty of Medicine, University of Belgrade, 11000 Belgrade, Serbia; drcoricvesna@gmail.com (V.C.); pekmezovic@orion.rs (T.P.); tatjana.simic@med.bg.ac.rs (T.S.); 3 Institute of Medical and Clinical Biochemistry, 11000 Belgrade, Serbia; 4 Institute of Epidemiology, 11000 Belgrade, Serbia

**Keywords:** colorectal cancer, survival, glutathione S-transferases

## Abstract

*Background and Objectives*: Despite improvements in screening programs, a large number of patients with colorectal cancer (CRC) are diagnosed in an advanced disease stage. Previous investigations imply that glutathione transferases (GSTs) might be associated with the development and progression of CRC. Moreover, the detoxification mechanism of oxaliplatin, which represents the first line of treatment for advanced CRC, is mediated via certain GSTs. The aim of this study was to evaluate the significance of certain *GST* genetic variants on CRC prognosis and the efficacy of oxaliplatin-based treatment. *Materials and Methods*: This prospective study included 523 patients diagnosed with CRC in the period between 2014 and 2016, at the Digestive Surgery Clinic, University Clinical Center of Serbia, Belgrade. Patients were followed for a median of 43.47 ± 17.01 months (minimum 1–63 months). Additionally, 109 patients with advanced disease, after surgical treatment, received FOLFOX6 treatment as a first-line therapy between 2014 and 2020. The *Kaplan*–*Meier* method was used to analyze cumulative survival, and the Cox proportional hazard regression model was used to study the effects of different *GST* genotypes on overall survival. *Results*: Individuals with the *GSTM1-null* genotype and the *GSTP1 IleVal+ValVal* (*variant)* genotype had significantly shorter survival when compared to referent genotypes (*GSTM1-active* and *GSTP1 IleIle*) (log-rank: *p* = 0.001). Moreover, individuals with the *GSTM1-null* genotype who received 5-FU-based treatment had statistically significantly shorter survival when compared to individuals with the *GSTM1-active genotype* (log-rank: *p* = 0.05). *Conclusions*: Both GSTM1-null and GSTP1 IleVal+ValVal (*variant*) genotypes are associated with significantly shorter survival in CRC patients. What is more, the *GSTM1-null* genotype is associated with shorter survival in patients receiving FOLOFOX6 treatment.

## 1. Introduction

Colorectal cancer (CRC) is the leading gastrointestinal malignancy worldwide, with an estimated 1.9 million new cases and 935,000 CRC-related deaths in 2020 [1]. The burden of CRC is on the rise globally, with Europe being one of the regions with the highest incidence and prevalence [2]. Despite improvements in screening programs, a great number of individuals are still being diagnosed in advanced disease stages [3]. The average 5-year survival rate of CRC patients remains at approximately 65%, which further emphasizes the need for establishing biomarkers of CRC progression [4].

Understanding the genetic foundation of CRC has been the focus of interest in numerous studies. The predominant type of CRC is adenocarcinoma, originating from the colon epithelium [5]. The process of carcinogenesis is stepwise and affected by multiple mutations through the previously described adenoma–carcinoma sequence [6]. Indeed, it has been suggested that CRC development is multifactorial in the majority of cases and has been attributed to a combination of sporadic mutations and environmental contributing factors [7]. Lifestyle habits, such as obesity, drinking, and smoking, are recognizable factors in CRC formation [8]. In contrast to rather well-known genetic and modifiable risk factors involved in CRC development, data regarding prognostic genetic factors are nevertheless scarce.

Earlier studies imply that glutathione transferases might be associated with both the development and the progression of CRC [9,10]. Glutathione transferases (EC 2.5.1.18), which are also known as glutathione S-transferases or GSTs, are enzymes with multiple functions and are engaged in a variety of activities, both catalytic and non-catalytic [11]. GSTs are generally considered the primary enzymes of the Phase II cellular detoxification system. To elaborate, GSTs are widely recognized for their ability to facilitate the nucleophilic addition of glutathione (GSH) to a diverse array of nonpolar compounds, whether they are of exogenous or endogenous origin [12]. These compounds contain electrophilic functional groups, which make the products more water-soluble, making it easier for Phase III enzymes of the cellular detoxification system to remove them from the cell [11]. Cytosolic GSTM1 and GSTT1 are especially significant in the biotransformation of polycyclic aromatic hydrocarbons, which can be detected in processed meat and cigarette smoke, which are well-recognized contributing factors to CRC development [13]. Aside from their function in biotransformation reactions, GSTs participate in processes of cellular survival, growth, and death. This is achieved via protein-protein interactions with particular signaling molecules. Specifically, GSTs have been demonstrated to exert a negative regulatory effect on protein kinases such as c-Jun NH2-terminal kinase (JNK1) and apoptosis signal-regulating kinase 1 (ASK1) [14]. GSTs are highly polymorphic in the general population, with polymorphisms leading to the modification of enzymatic activity [15]. Precisely, both deletional and single-nucleotide polymorphisms (SNPs) are responsible for the complete absence or alteration of enzyme activity [15]. Regarding CRC, several studies have implied that GSTs might play a role, not only in the development but also in CRC progression [16,17,18]. However, data regarding the potential role of different *GST* genetic variants are inconsistent and require further investigation [18].

The first-line therapy in the treatment of patients with advanced colorectal cancer includes the FOLFOX6 regimen, which represents a combination of 5-fluorouracil/leucovorin (5FU/LV) and oxaliplatin [19]. Oxaliplatin expresses its chemotherapeutic effects by generating DNA adducts of platinum derivate with the DNA base, further resulting in programmed cellular death [17]. Furthermore, oxaliplatin-based chemotherapy is shown to contribute to oxidative stress. Since the detoxification mechanism of oxaliplatin is mediated via certain GSTs, their polymorphic expression could lead to an alteration in the response to applied chemotherapeutics [20]. Bearing this in mind, it might be speculated that *GST* polymorphisms might serve as potential biomarkers in the prediction of oxaliplatin-based treatment efficacy [21].

Taking into consideration that *GST* polymorphisms might play an important role in CRC progression but could also affect the treatment outcome, the aim of this study was to evaluate the possible role of certain *GST* genetic variants in CRC prognosis, as well as whether it might affect the efficacy of oxaliplatin-based treatment in these patients.

## 2. Materials and Methods

### 2.1. Data Source and Patient Selection

Patients included in this study had clinically (via colonoscopy) and histopathologically verified presence of CRC. Exclusion criteria were a previous history of another malignancy, including recurrent CRC, or a desire of the patient to no longer participate in the study. The CRC histopathological confirmation was consistent with the TNM and Dukes classification [22].

### 2.2. Study Design

This prospective study included 523 patients diagnosed with CRC in the period between 2014 and 2016, at the Digestive Surgery Clinic, University Clinical Center of Serbia, Belgrade. Patients were followed for a median of 43.47 ± 17.01 months (minimum 1–63 months). Data regarding age, sex, body mass index, and smoking status, as well as primary localization of the carcinoma, TNM stage, differentiation, and perineural and lymphovascular invasion, were collected.

In our cohort, 109 patients with advanced disease received FOLFOX6 treatment as the first-line therapy after surgical treatment between 2014 and 2020. FOLFOX6 treatment consisted of 2 h infusion of oxaliplatin (100 mg/m^2^), 2 h infusion of leucovorin (400 mg/m^2^), and a bolus of 5 FU (400 mg/m^2^) followed by 5 FU (2500 mg/m^2^) over 46 h in a continuous infusion [23]. The patients received this treatment every two weeks unless the disease progressed.

A structured questionnaire developed at the Institute of Epidemiology, Faculty of Medicine, University in Belgrade, was used for collecting patients’ data regarding socio-demographic factors. This study was approved by the Institutional Ethical Board (approval number 56-6, University Clinical center of Serbia) and performed according to the principles of the Helsinki Declaration. Written consent was obtained prior to inclusion in the study.

Overall survival (OS) was expressed as the time from surgery until the date of the last follow-up (1 June 2020) or the time of death. Follow-up data were obtainable for 471 patients with CRC, while the contact information for 52 patients (9%) was lost. The treatment response was evaluated according to the response evaluation criteria in solid tumors (RECIST) guidelines [24]. In addition, after every three cycles of FOLFOX6 treatment, each patient underwent a diagnostic algorithm consisting of an abdominal ultrasound and a CT chest–abdomen–pelvis protocol.

### 2.3. Peripheral Blood Collection and Assay

The peripheral blood of patients with CRC was used for DNA isolation by QIAamp DNA Blood Mini Kit (Qiagen, #51306, Chatsworth, CA, USA) according to the protocol of the manufacturer. As described by Abdel-Rahman et al., multiplex PCR was utilized to identify homozygous deletions of *GSTM1* and *GSTT1* [25]. For the detection of *GSTA1* C69T (rs3957357) SNP, in accordance with Ping et al., the PCR-restriction fragment length polymorphism (RFLP) approach using the Eam1104I (Thermo Fisher Scientific, Waltham, MA, USA) restriction enzyme was used [26]. The representative findings analyzed on the appropriate agarose gels are presented in Supplementary Figures S1 and S2. The Applied Biosystems TaqMan^®^ Drug Metabolism Genotyping assay (Life Technologies, Applied Biosystems, Carlsbad, CA, USA, assay ID: C 3,237,198 20) was utilized to analyze the *GSTP1* Ile105Val (rs1695) SNP according to the manufacturer’s protocols.

### 2.4. Statistical Analysis

Statistical analysis was performed using SPSS ver. 20.0 (IBM Corporation, Armonk, NY, USA) [27]. The genotype distribution was assessed for the presence of deviation from Hardy–Weinberg equilibrium. The cumulative survival estimation was based on the *Kaplan–Meier* method. The Cox proportional hazard regression model was used to study the effects of different *GST* genotypes on overall survival. In addition, models have been adapted by covariates. Model 1 was adjusted to sex and age, Model 2 to covariates of Model 1 as well as obesity and smoking, Model 3 to covariates of Model 2 and FOLFOX6 treatment, and Model 4 to tumor characteristics: localization and differentiation. The results were regarded as statistically significant if the *p*-value was ≤0.050.

## 3. Results

Selected patients’ characteristics are outlined in Table 1. Among 471 CRC patients with successfully acquired follow-up data, there were 131 deaths (28%) during the follow-up period. A statistically significant difference in terms of patients’ outcomes was observed regarding the TNM classification and grade of the primary tumor (*p* = 0.001 and *p* = 0.005, respectively). Namely, the frequency of the lethal outcome was higher in patients with higher-stage and -grade colorectal cancer. On the other hand, no association was observed regarding colorectal cancer side localization. Of note, the predominant localization of CRC was the rectum (55%), and, in the majority of cases, the tumor was well-differentiated (77%), while the T3 stage (49%) was the most frequent one.

### 3.1. GST Genotypes and Survival

Table 2 represents the possible association between different *GST* genotypes and outcomes in patients with colorectal carcinoma during the follow-up period.

As presented, there was a statistically significant difference in patients’ outcomes depending on the presence of different *GST* genetic variants (*p* = 0.001 and *p* = 0.009, respectively). Precisely, individuals with *GSTM1-null* and *GSTP1 IleVal+ValVal* (*variant)* genotypes had significantly higher frequencies of lethal outcomes when compared to carriers of *GSTM1*-active and *GSTP1 IleIle* (*referent*) genotypes. Regarding the other two investigated polymorphisms, no significant association was observed between *GSTT1* and *GSTA1* genetic variants and disease outcomes in CRC patients.

### 3.2. Effect of GST Polymorphisms on CRC Patients’ Overall Survival

The *Hardy–Weinberg* equilibrium (HWE) was assessed indicating a deviation only for *GSTP1* rs1695 (the chi-squared value of 6.029 and the chi-squared test *p*-value of 0.014). *Cox* regression analysis confirmed the significance of both *GSTM1-null* and *GSTP1 IleVal+ValVal* (*variant)* genotypes as independent prognostic factors for increased overall mortality in patients with colorectal cancer. When analyzed in four different models, both *GSTM1-null* and *GSTP1 IleVal+ValVal* (*variant)* genotypes showed significant multivariable-adjusted HR (Table 3, Table 4, Table 5 and Table 6), while regarding other *GST* genotypes, the obtained results were not statistically significant (*p* > 0.05).

Namely, taking into consideration covariates that might contribute to CRC patients’ prognosis (age, sex, lifestyle habits, treatment, and tumor characteristic), *GSTM1-null* and *GSTP1 IleVal+ValVal* (*variant)* exhibited a significant prognostic effect in all assessed models, indicating a higher risk of mortality in carriers of these genotypes. Indeed, when the analysis of the effect of GST genetic variants on CRC patients’ overall survival was conducted using Model 1 (adjustment to age and sex), the obtained results clearly indicated that the risk of mortality was significantly higher (HR 1.81, 95% CI 1.22–2.68, *p* = 0.003) in individuals lacking the GSTM1 protein when compared to those with the intact protein presence. In this line, the risk of mortality was 1.53 times significantly higher (95% CI 0.67–2.46) in carriers of the *GSTP1*-*variant* (*IleVal* or *ValVal*) genotype in comparison to carriers of the *GSTP1*-*referent* genotype (*p* = 0.048). However, such an effect was not observed in the case of either *GSTT1* or *GSTA1* genotypes (Table 3).

The next model applied in the analysis, Model 2, apart from adjustments to age and sex, also included known CRC risk factors, such as smoking and BMI. The prognostic effect of *GST* genotypes recognized using Model 1 remained. Namely, the *GSTM1-null* genotype increased the risk of mortality by 1.79 times (*p* = 0.04), while the *GSTP1-variant* genotype was associated with a 1.57 times higher mortality risk in CRC patients (*p* = 0.035) in comparison to the reference *GSTM1-active* and *GSTP1-referent IleIle* genotypes, respectively (Table 4).

The statistical model presented in Table 5, Model 3, is adjusted to covariates of Model 2, but also includes information regarding the applied FOLFOX6 treatment in patients with colorectal cancer. Similarly to results obtained in the two previous models, genetic variations in *GSTM1* and *GSTP1* exhibited a prognostic effect in terms of mortality risk in CRC patients, while the observed effect was lacking in cases of *GSTT1* and *GSTA1* polymorphisms.

Last, but not least, Model 4 included adjustments to tumor characteristics (colorectal cancer localization and differentiation) in the analysis of the prognostic effect of *GST* genetic variations on the risk of overall mortality in CRC patients (Table 6). The obtained results resemble the results from the analysis using previous models, further highlighting the role of *GSTM1* and *GSTP1* polymorphisms as possible determinants of mortality risk in patients with colorectal cancer (HR 1.89, *p* = 0.001 and HR 1.50, *p* = 0.046, respectively).

### 3.3. The Relevance of GST Polymorphisms in the Overall Survival of CRC Patients

**Figure 1:**
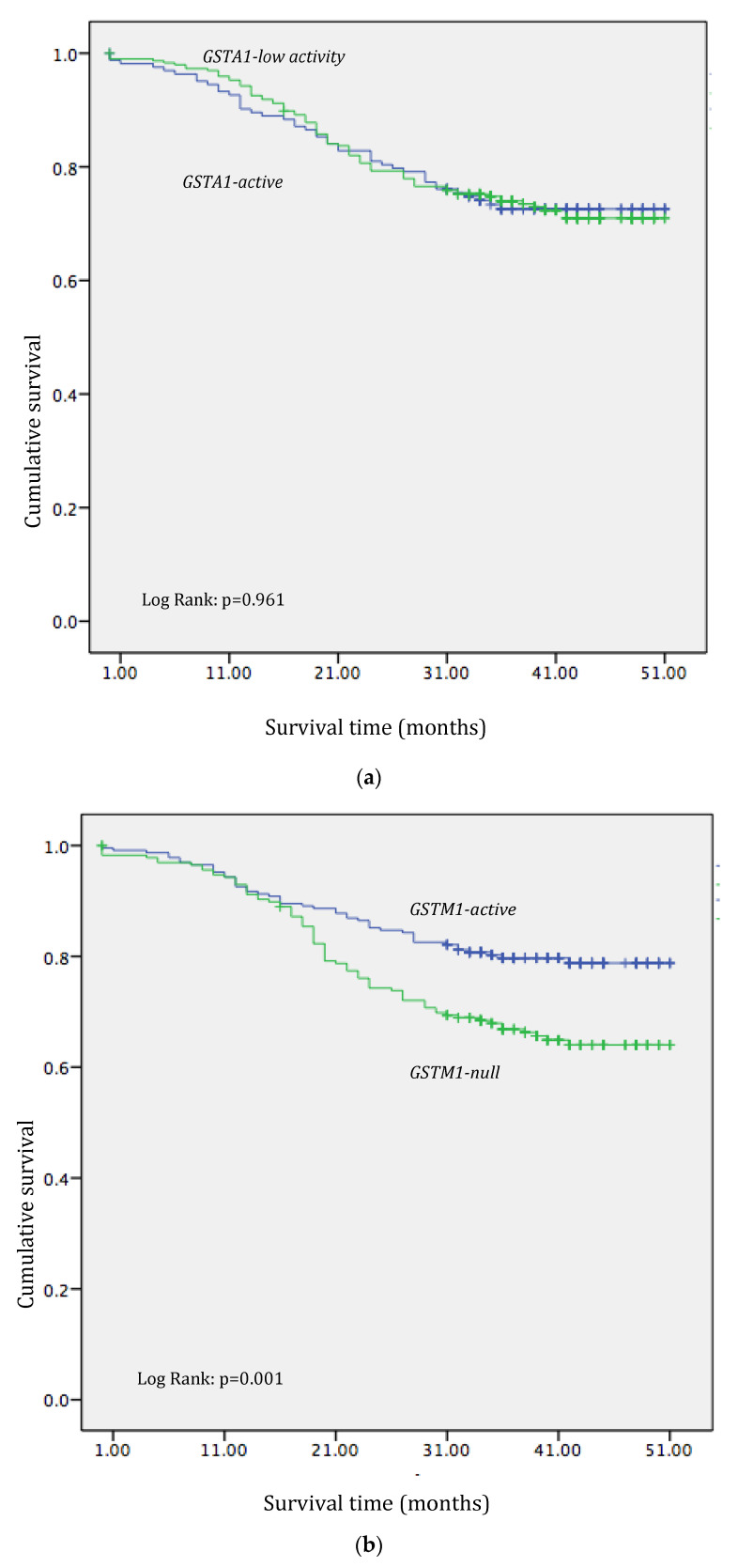

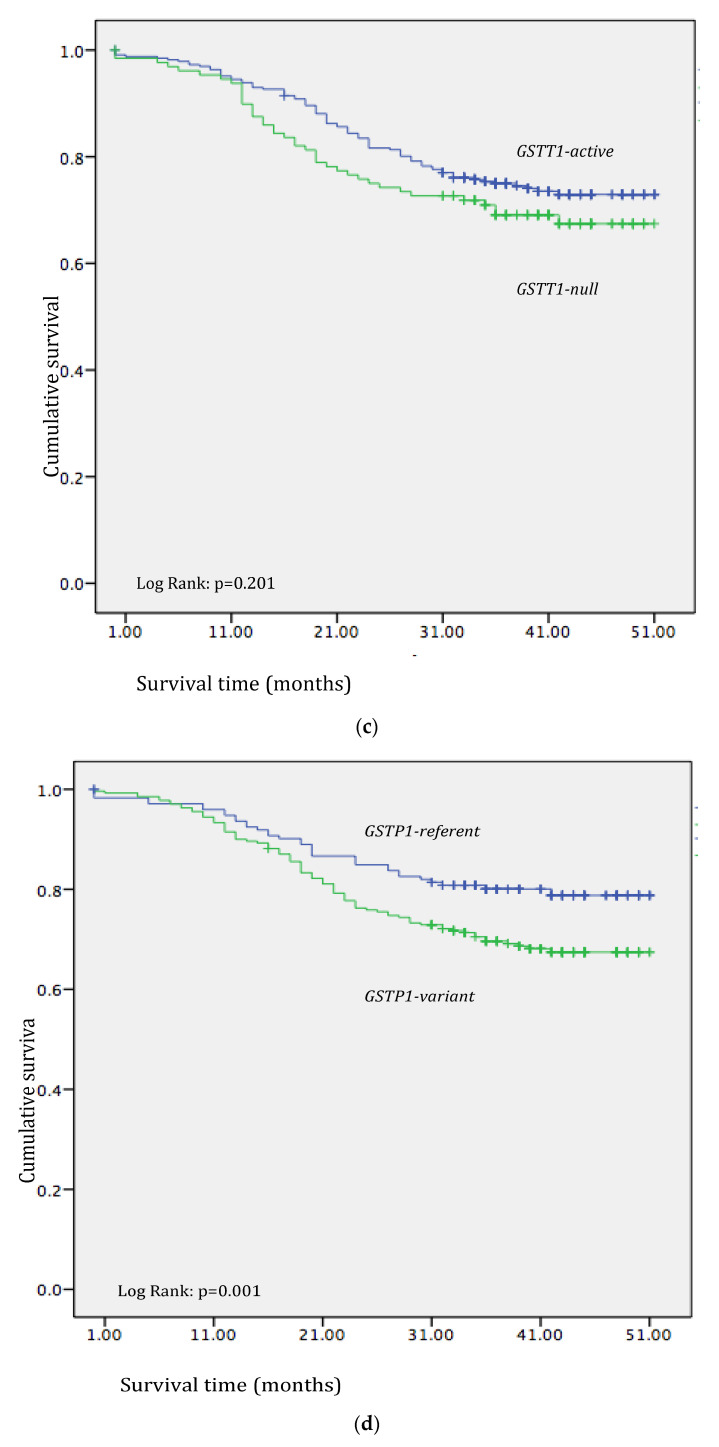
Overall survival of individuals with CRC with respect to different GST genotypes (**a**) *Kaplan–Meier* survival curves according to *GSTA1* polymorphism; *Low activity* if one active allele is present; (**b**) *Kaplan–Meier* survival curves according to *GSTM1* polymorphism; *Active* if one active allele is present; *Null* if no active allele is present (**c**) *Kaplan–Meier* survival curves according to *GSTT1* polymorphism; *Active* if one active allele is present; *Null* if no active allele is present (**d**) *Kaplan–Meier* survival curves according to *GSTP1* polymorphism; *Variant* if one Val allele is present.

*Kaplan*–*Meier* analysis showed statistically significantly shorter overall survival in individuals with the *GSTM1-null* genotype when compared to carriers of the GSTM1-active genotype (log-rank: *p* = 0.001, Figure 1b). Additionally, the *Kaplan–Meier* analysis showed statistically significant shorter overall survival in carriers of the *GSTP1 IleVal+ValVal* (*variant)* genotype in comparison to individuals with the *GSTP1 IleIle (referent)* genotype (log-rank: *p* = 0.001, Figure 1d). However, no statistically significant effect of either *GSTA1* or *GSTT1* polymorphisms was observed in terms of overall survival (Figure 1a,c) in CRC patients (*p* > 0.05).

### 3.4. Effects of GST Polymorphisms on the Overall Survival of CRC Patients on 5-FU-Based Treatment

**Figure 2:**
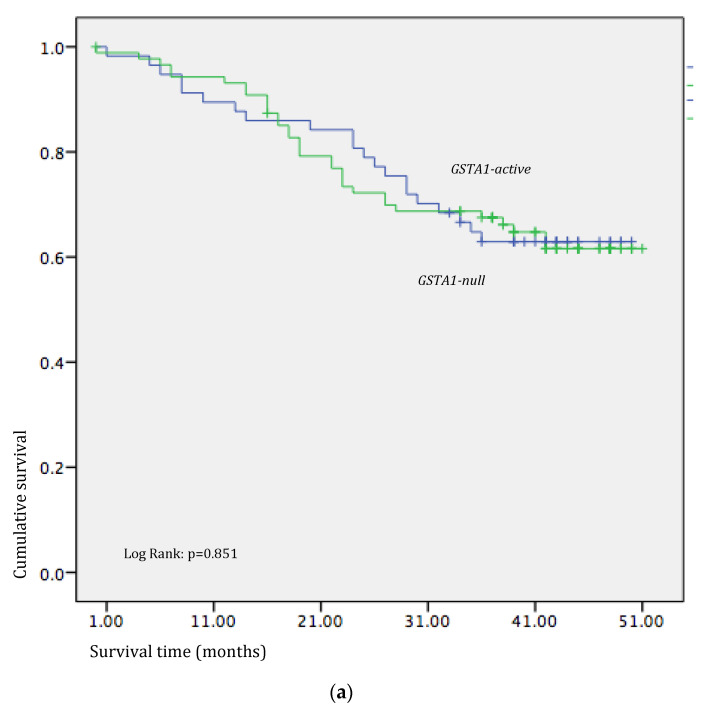

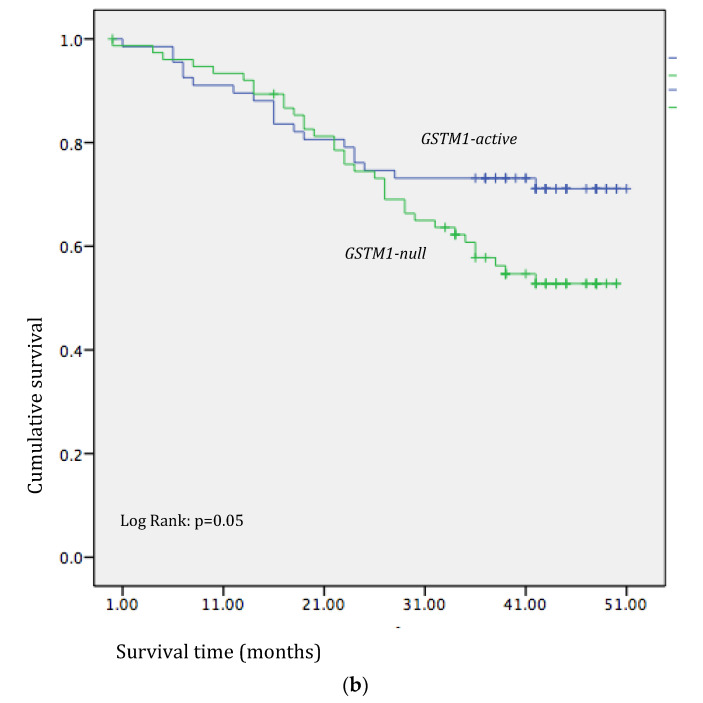

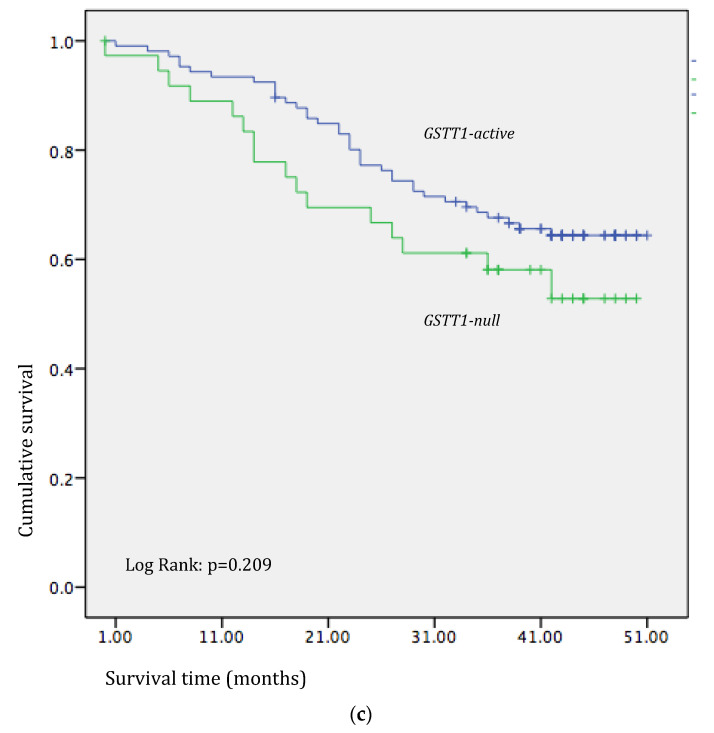

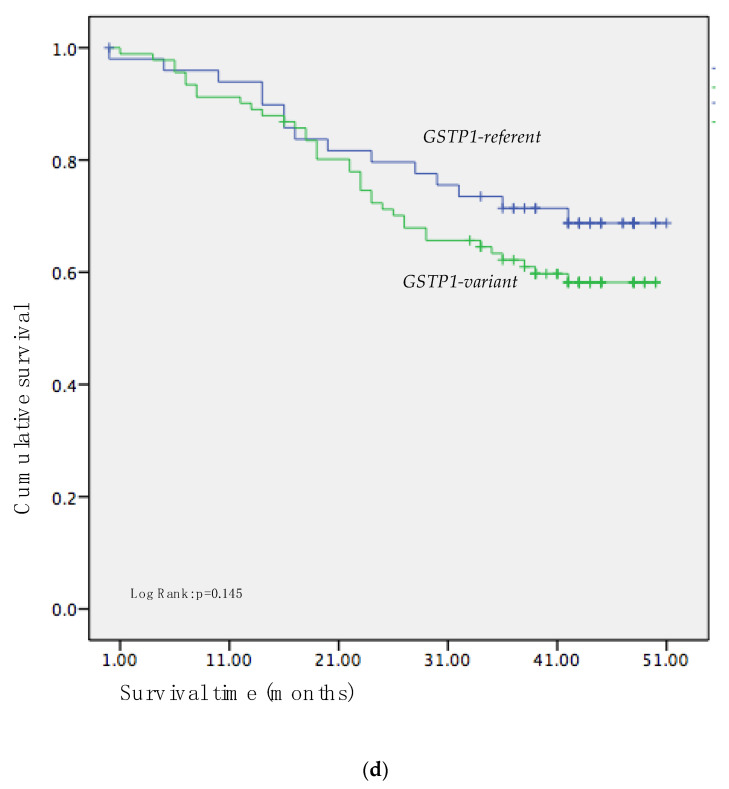
Effects of *GST* polymorphisms on overall survival of CRC patients on 5-FU-based treatment. (**a**) *Kaplan–Meier* survival curves according to *GSTA1* polymorphism; *Low activity* if one active allele is present; (**b**) *Kaplan–Meier* survival curves according to *GSTM1* polymorphism; *Active* if one active allele is present; *Null* if no active allele is present (**c**) *Kaplan–Meier* survival curves according to *GSTT1* polymorphism; *Active* if one active allele is present; *Null* if no active allele is present; (**d**) *Kaplan–Meier* survival curves according to *GSTP1* polymorphism; *Variant* if one *Val* allele is present.

In the next step, we further analyzed the potential significance of certain *GST* genetic variants on the efficacy of oxaliplatin-based treatment in CRC patients since GSTs participate in the metabolism of the applied drug. Interestingly, *Kaplan–Meier* analysis showed statistically significant shorter overall survival in individuals with the *GSTM1-null* genotype who received 5-FU-based treatment when compared to individuals with the *GSTM1-active genotype* (log-rank: *p* = 0.05, Figure 2b), which was not observed for other investigated genotypes (log-rank: *p >* 0.05, Figure 2a,c,d). Surprisingly, in our group of patients with colorectal cancer, no association was observed in the case of GSTP1, which is the class of glutathione transferases known for its role in drug metabolism, as well as the effect on chemoresistance development, which has previously been associated with the metabolism of platinum derivatives.

## 4. Discussion

In this study, we have assessed the effect of four common *GST* polymorphisms in terms of CRC prognosis. Our results suggested that individuals with the *GSTM1-null* genotype, as well as individuals with the GSTP1 IleVal+ValVal *(variant)* genotype, have significantly shorter overall survival in comparison to individuals with the corresponding *referent genotypes.* In addition, when the association between different GST genetic variants and overall mortality was analyzed by applying the specified and adjusted statistical Models, GSTM1-null and GSTP1-*variant* genotypes were recognized as independent prognostic factors for increased overall mortality. Furthermore, carriers of the GSTM1-null genotype who underwent treatment with FOLFOX6 had significantly shorter *overall* survival compared to individuals with the GSTM1-active genotype.

For years, the role of GST genetic polymorphisms in patients with CRC has drawn researchers’ attention worldwide. The rationale for this is the fact that environmental factors are recognized as significant contributing factors in CRC development and progression, while GSTs are known for their role in xenobiotic detoxification and inactivation. [13,28]. However, available data regarding a possible association between GST polymorphisms and CRC development and progression are still quite controversial [16,27,29].

Glutathione transferase M1 is among the most extensively studied *GST* polymorphisms associated with cancer development and progression in general. Deletion polymorphism of the *GSTM1* gene leads to the complete absence of protein, meaning a complete lack of enzyme activity, which could further result in increased susceptibility to carcinoma development in carriers of the *GSTM1-null* genotype [30]. Although several meta-analyses have investigated the risk of CRC development in carriers of *GSTM1-active* vs. *GSTM1-null* genotypes, the obtained results are still debatable [31,32]. Ethnicity, as well as geographical region, could be among the main factors influencing these conflicting data [33]. On the other hand, studies regarding the possible role of the *GSTM1* genotype as a prognostic biomarker in patients with CRC are scarce. Csejtei et al. found that patients with the *GSTM1-null* genotype have significantly lower survival when compared to individuals with the *GSTM1-active* genotype, which is in accordance with our study [34]. In this line, Liu et al. concluded that the *GSTM1-null* genotype is associated with shorter overall survival in Caucasian patients with CRC [31]. Furthermore, Feng et al. recently suggested that *GSTM1* might be associated with outcomes in CRC patients [35]. All these results justify the researchers’ focus on investigating the differential roles of GSTM1 in colorectal cancer.

Gene deletion of another glutathione transferase, *GSTT1,* also results in the complete lack of this protein in the gastrointestinal tract, significantly affecting and disabling the detoxification ability in individuals carrying the *GSTT1-null* genotype. For that reason, the *GSTT1-null* genotype is another of the most frequently investigated *GST* genotypes [23,24,25]. Although the *GSTT1-null* genotype has so far been associated with CRC susceptibility in Caucasians, data regarding its effect on overall survival in patients with CRC are not so abundant [36]. Indeed, Liu et al. conducted a meta-analysis that included 13 articles on the association between *GSTT1* polymorphism and gastric or colorectal cancer outcomes and found a lack of data when it comes to overall survival in Caucasian patients with CRC, which is in agreement with our results [31].

An additional focus of our study was the assessment of *GSTA1* polymorphism in CRC, considering that it has been studied far less than other GST genetic variants. One of the first studies investigating the association between *GSTA1* polymorphism and CRC development was that of Martinez et al., which showed that GSTA1 polymorphic expression does not influence CRC susceptibility [37]. Although meta-analyses regarding *GSTA1* genetic variability and susceptibility to CRC development were conducted, studies regarding the association with the outcome in CRC patients are lacking [9,10]. Our results suggest that there is no significant effect of *GSTA1* rs 3,957,357 genetic variation on overall survival in patients with CRC.

Due to its role in chemoresistance, GSTP1 is by far the most extensively investigated glutathione transferase in cancers. Namely, GSTP1 is shown to be overexpressed in numerous tumors, including CRC [38], implying that it plays an important role in the complex processes of carcinogenesis [10]. However, when it comes to the results on the association between *GSTP1* genotypes and the survival of CRC patients, the available data seem rather conflicting. In the recent study by Rodriguez-Fleming et al. [16], *GSTP1* polymorphism was not associated with the survival of patients with CRC, which is not in accordance with our results. Indeed, we have found that the presence of different *GSTP1* genetic variants affects survival in CRC patients in terms of shorter overall survival in carriers of the GSTP1 IleVal+ValVal (*variant)* genotype when compared to individuals with the *GSTP1 IleIle (referent)* genotype. The possible explanation for this discrepancy in our findings may be due to the fact, which was previously mentioned, that the distribution of different *GSTP1* genotypes might vary between people of different geographical and ethnic origins. Furthermore, in the aforementioned study, the sample size might be of influence, as it consisted of around 200 individuals as opposed to our cohort, which included over 500 patients.

Since previous findings have indicated that cancer cells in general might have a higher level of GST expression, which could potentially influence the detoxification of anticancer therapy [39], this seemed significant in terms of colorectal cancer treatment. FOLFOX6 treatment represents the first-line therapy for CRC in patients with advanced disease and the response rate varies at around 40% [40]. Considering the high extent of response variability among patients, the meta-analysis by Shahnam et al. evaluated the role of different genetic polymorphisms in patients’ responses to oxaliplatin-based therapy, as well as in the survival of patients with CRC [41]. Among the 32 studies that were included in this investigation, only 3 analyzed the presence of *GSTM1*, either in Asian or Caucasian populations, while the data regarding the overall survival in CRC patients were actually lacking. However, the results of McLeod et al. imply that individuals with the *GSTM1-null* genotype had significantly higher numbers of adverse effects and lower survival rates [42]. Additionally, although statistical significance was lacking, the results of Boige et al. indicated shorter overall survival in patients with the GSTM1-null genotype [43]. The results of our study, which suggest that individuals with the *GSTM1-null* genotype who received 5-FU-based treatment had significantly shorter overall survival compared to individuals with the *GSTM1-active* genotype, are in concordance with both mentioned studies [42,43].

Another GST polymorphism that was analyzed in CRC patients receiving FOLFOX6 treatment can be seen in the study of Stoehlamacher et al., who found that the *GSTP1 IleVal* genotype is associated with increased survival in patients receiving this treatment [44]. This result is not in agreement with our results, possibly since, in our study, we analyzed the presence of at least one variant GSTP1 allele or, more precisely, either the *IleVal* or *ValVal* genotype. Although certain traditional anti-cancer medications like cisplatin can be affected by GST expression and deactivated through a process called conjugation with glutathione, there are also other potential mechanisms through which GSTs could contribute to the development of resistance to anti-cancer medications [45]. Indeed, a multitude of anti-cancer substances trigger the process of apoptosis by activating the kinase pathway, particularly involving JNK and p38. What is more, cisplatin is a medication that relies on JNK activity to achieve its maximum cytotoxic effect in a way that the suppression of the JNK signaling pathway results in a reduction in cisplatin-induced cell death [12]. However, the impact of polymorphic expression of GSTP1 on the apoptosis that is dependent on JNK1 has not been explained so far. There has only been one study that has demonstrated that the *GSTP1 Val* allele is a more effective JNK1 inhibitor and thus has a stronger antiapoptotic impact than the *wild-type Ile* allele [46].

Several constraints of this investigation necessitate attention. Loss to follow-up can introduce bias into the assessment of association. Furthermore, in order to gain further insight into the impact of GST polymorphisms on overall survival, it would be advantageous to explore its potential correlation with cancer-specific survival in a broader group of individuals. Additionally, conducting future research on a significantly larger study group, which would include individuals with different ethnicities and geographic origins to investigate the collective impact of GST polymorphisms on the prognosis of colorectal cancer patients, would be highly helpful.

## 5. Conclusions

This study supports the hypothesis that *GST* polymorphisms might have an effect on the overall survival of patients with colorectal cancer. The *GSTM1-null* genotype and the GSTP1 IleVal+ValVal *(variant)* genotype are found to be associated with significantly shorter survival in CRC patients. Additionally, the *GSTM1-null* genotype affects the survival of patients receiving FOLFOX6 treatment. Further studies are necessary to shed more light on the supposed role of GST genetic variants in the prognosis of patients with colorectal cancer.

## Figures and Tables

**Table 1:** Available patient clinical characteristics.

	Living, n%	Deceased, n%	*p*-Value
**Sex**			
Male	203 (70)	86 (30)	0.235
Female	137 (75)	45 (25)	
**Age** (mean ± SD)	62.42 ± 10.47	62.71 ± 13.09	0.815
**Localization**			
Left side	65 (78)	23 (22)	0.697
Right side	275 (73)	108 (27)	
**TNM**			
I	123 (93)	9 (7)	0.001
II	106 (88)	14 (12)	
III	86 (58)	62 (42)	
IV	25 (36)	46 (64)	
**Grade**			
Well differentiated	267 (76)	84 (24)	0.005
Moderately differentiated	61 (60)	38 (40)	
Poorly differentiated	12 (57)	9 (43)	

**Table 2:** *GST* genotype distribution in living and deceased patients with colorectal cancer.

*GST* Genotype	Living, n%	Deceased, n%	*p*-Value
*GSTM1*			
*active ^a^*	182 (80)	47 (20)	0.001
*null ^b^*	150 (66)	79 (34)	
*GSTT1*			
*null ^b^*	89 (69)	41 (31)	0.246
*active ^a^*	243 (74)	89 (26)	
*GSTA1* (rs 3957357)			
*CC (active)*	120 (73)	44 (27)	0.913
*CT+TT (low activity) ^c^*	215 (72)	82 (28)	
*GSTP1* (rs1695)			
*IleIle (referent)*	139(80)	35 (20)	0.009
*IleVal+ValVal (variant) ^d^*	185 (68)	86 (32)	

*^a^ Active*, at least one allele present *^b^ Null*, none of the alleles are present; *^c^ Variant*, at least one *Val* allele present; *^d^ Low activity* at least one *T* allele present; the data represent the number of successful genotypisations.

**Table 3:** The prognostic effect of *GST* genotypes on the risk of overall mortality in CRC patients (Model 1).

Model 1	
HR (95% CI)	*p*-Value
Risk of mortality in carriers of *GSTM1-null ^a^* genotype compared to carriers of *GSTM1-active ^b^* genotype	
1.81 (1.22–2.68)	0.003
Risk of mortality in carriers of *GSTT1-null ^a^* genotype compared to carriers of *GSTT1-active ^b^* genotype	
1.24 (0.82–1.87)	0.301
Risk of mortality in carriers of *GSTA1-low activity ^c^* genotype compared to carriers of *GSTA1-active ^b^* genotype	
1.03 (0.69–1.53)	0.873
Risk of mortality in carriers of *GSTP1-variant ^d^* genotype compared to carriers of *GSTP1-referent* genotype	
1.53 (0.67–2.46)	0.048

Model 1 is adjusted to age and sex; *^a^ Active*, if at least one active allele is present; *^b^ Null* if no active alleles are present; *^c^ Low activity*, if at least one *T* allele is present. *^d^ Variant*, if at least one *Val* allele is present; HR, hazard ratio; CI, confidence interval.

**Table 4:** The prognostic effect of *GST* genotypes on the risk of overall mortality in CRC patients (Model 2).

Model 2	
HR (95% CI)	*p*-Value
Risk of mortality in carriers of *GSTM1-null ^a^* genotype compared to carriers of *GSTM1-active ^b^* genotype	
1.79 (1.21–2.65)	0.004
Risk of mortality in carriers of *GSTT1-null ^a^* genotype compared to carriers of *GSTT1-active ^b^* genotype	
1.24 (0.82–1.88)	0.300
Risk of mortality in carriers of *GSTA1-low activity ^c^* genotype compared to carriers of *GSTA1-active ^b^* genotype	
1.00 (0.67–1.50)	0.986
Risk of mortality in carriers of *GSTP1-variant ^d^* genotype compared to carriers of *GSTP1-referent* genotype	
1.57 (1.03–2.39)	0.035

Model 2 is adjusted to the covariates of Model 1 and known CRC risk factors (smoking, BMI); *^a^ Active*, if at least one active allele is present; *^b^ Null* if no active alleles are present; *^c^ Low activity*, if at least one *T* allele is present. *^d^ Variant*, if at least one *Val* allele is present; HR, hazard ratio; CI, confidence interval.

**Table 5:** The prognostic effect of *GST* genotypes on the risk of overall mortality in CRC patients (Model 3).

Model 3	
HR (95% CI)	*p*-Value
Risk of mortality in carriers of *GSTM1-null ^a^* genotype compared to carriers of *GSTM1-active ^b^* genotype	
1.89 (1.21–2.65)	0.001
Risk of mortality in carriers of *GSTT1-null ^a^* genotype compared to carriers of *GSTT1-active ^b^* genotype	
1.25 (0.85–1.84)	0.250
Risk of mortality in carriers of *GSTA1-low activity ^c^* genotype compared to carriers of *GSTA1-active ^b^* genotype	
1.02 (0.70–1.48)	0.918
Risk of mortality in carriers of *GSTP1-variant ^d^* genotype compared to carriers of *GSTP1-referent* genotype	
1.47 (0.99–2.20)	0.050

Model 3 is adjusted to covariates of Model 2 and FOLFOX6 treatment; *^a^ Active*, if at least one active allele present; *^b^ Null* if no active alleles present; *^c^ Low activity*, if at least one *T* allele present. *^d^ Variant*, if at least one *Val* allele present; HR, hazard ratio; CI, confidence interval.

**Table 6:** The prognostic effect of *GST* genotypes on the risk of overall mortality in CRC patients (Model 4).

Model 4	
HR (95% CI)	*p*-Value
Risk of mortality in carriers *GSTM1-null ^a^* genotype compared to carriers of *GSTM1-active ^b^* genotype	
1.89 (1.31–2.74)	0.001
Risk of mortality in carriers *GSTT1-null ^a^* genotype compared to carriers of *GSTT1-active ^b^* genotype	
1.26 (0.86–1.84)	0.245
Risk of mortality in carriers *GSTA1-low activity ^c^* genotype compared to carriers of *GSTA1-active ^b^* genotype	
1.02 (0.70–1.49)	0.902
Risk of mortality in carriers *GSTP1-variant ^d^* genotype compared to carriers of *GSTP1-referent* genotype	
1.50 (1.01–2.24)	0.046

Model 4 is adjusted to covariates of tumor localization and differentiation; *^a^ Active*, if at least one active allele is present; *^b^ Null* if no active alleles are present; *^c^ Low activity*, if at least one *T* allele is present. *^d^ Variant*, if at least one *Val* allele is present; HR, hazard ratio; CI, confidence interval.

## Data Availability

The data supporting the reported results can be found upon request in the form of datasets available at the Clinic of Gastroenterohepatology, University Clinical Centre of Serbia and at the Institute of Medical and clinical Biochemistry, Faculty of Medicine University of Belgrade.

## Supplementary Materials

The following supporting information can be downloaded at: https://www.mdpi.com/article/10.3390/medicina60040553/s1, Figure S1. 2% agarose gel electrophoresis: PCR genotyping for GSTM1 and GSTT1 polymorphisms. Lane 1 comprises DNA marker (ladder). Lanes 3, 5, 7, 8, 11 comprise PCR products of patients with the GSTT1-active/GSTM1-active genotype; Lanes 2, 4, 10 comprise PCR products of patients with the GSTT1-active/GSTM1-null genotype; Lane 6 and 9 indicate patients with GSTT1-null/GSTM1-null genotype; CYP1A1 was used as housekeeping gene (present in lanes 2–11); Figure S2. 3% agarose gel electrophoresis: PCR-RFLP restriction products of the *GSTA1* gene. Lanes 1, 2, 4, 8, 10, 11, 14 comprise PCR products of patients with the *GSTA1**CC genotype; Lanes 5, 6, 7, 9, 13 comprise PCR-RFLP restriction products of patients with the *GSTA1**CT genotype; Lanes 3 and 12 comprise RFLP-PCR restriction products of patients with *GSTA1**TT genotype. Lane 15 comprises DNA marker (ladder). The Applied Biosystems TaqMan^®^ Drug Metabolism Genotyping assay (Life Technologies, Applied Biosystems, USA, assay ID: C 323719820) was utilized to analyze the *GSTP1* Ile105Val (rs1695), and was assessed using *Eppendorf Mastercycler ep^®^ realplex*, according to the manufacturer protocol.
