# Supplementary material for: GSTM1 and GSTP1 Polymorphisms Affect Outcome in Colorectal Adenocarcinoma
Source: Medicina (Kaunas). 2024 Mar 28;60(4):553. doi: 10.3390/medicina60040553 (PMC11052438; doi:10.3390/medicina60040553)
Supplement: Supplementary file 1 [file medicina-60-00553-s001.zip › medicina-2895073-supplementary.pdf]

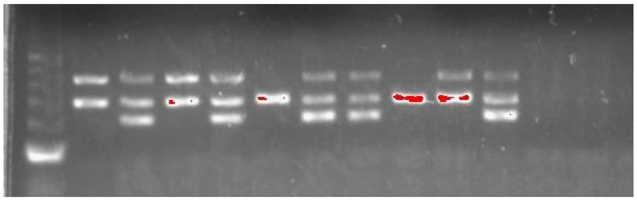

Supplementary Figure S1. 2% agarose gel electrophoresis: PCR genotyping for *GSTM1* and *GSTT1* polymorphisms. Lane 1 comprises DNA marker (ladder). Lanes 3, 5, 7, 8, 11 comprise PCR products of patients with the *GSTT1-active*/*GSTM1-active* genotype; Lanes 2, 4, 10 comprise PCR products of patients with the *GSTT1-active*/*GSTM1-null* genotype; Lane 6 and 9 indicate patients with *GSTT1-null*/*GSTM1-null* genotype; *CYP1A1* was used as housekeeping gene (present in lanes 2-11).

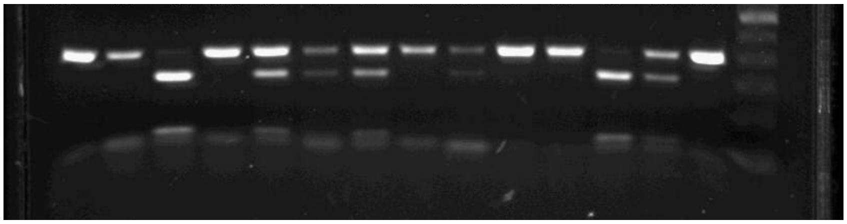

Supplementary Figure S2. 3% agarose gel electrophoresis: PCR-RFLP restriction products of the *GSTA1* gene. Lanes 1, 2, 4, 8, 10, 11, 14 comprise PCR products of patients with the *GSTA1**CC genotype; Lanes 5, 6, 7, 9, 13 comprise PCR-RFLP restriction products of patients with the *GSTA1**CT genotype; Lanes 3 and 12 comprise RFLP-PCR restriction products of patients with *GSTA1**TT genotype. Lane 15 comprises DNA marker (ladder).
